# Supplementary material for: Low-Cost Zinc–Alginate-Based Hydrogel–Polymer Electrolytes for Dendrite-Free Zinc-Ion Batteries with High Performances and Prolonged Lifetimes
Source: Polymers (Basel). 2022 Dec 31;15(1):212. doi: 10.3390/polym15010212 (PMC9823846; doi:10.3390/polym15010212)
Supplement: Supplementary file 1 [file polymers-15-00212-s001.zip › polymers-2058904-supplementary.pdf]

Supplementary Information

Low-cost Zinc-Alginate-based Hydrogel-Polymer Electrolyte for Dendrite-free Zinc-Ion Batteries with High Performances and Prolonged Lifetime

Zhuoyuan Zheng, Haichuan Cao, Wenhui Shi, Chunling She, Xianlong Zhou, Lili Liu and Yusong Zhu*

School of Energy Science and Engineering, Nanjing Tech University, Nanjing 211816, Jiangsu Province, China
* Correspondence: zhuys@njtech.edu.cn (Y.Z.)

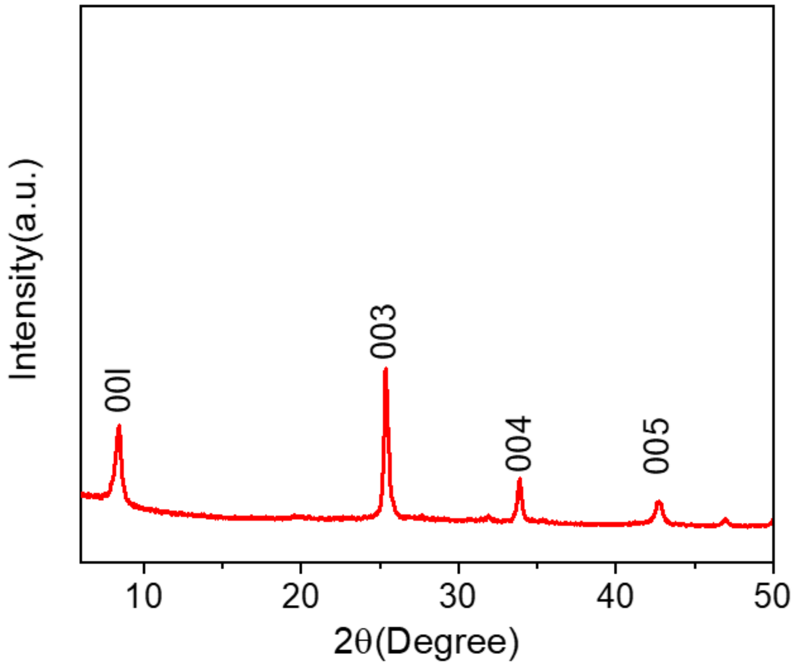

Figure S1. The XRD pattern of the $Ca_{0.24}V_2O_5 \bullet 0.83H_2O$ (CVO) cathode.

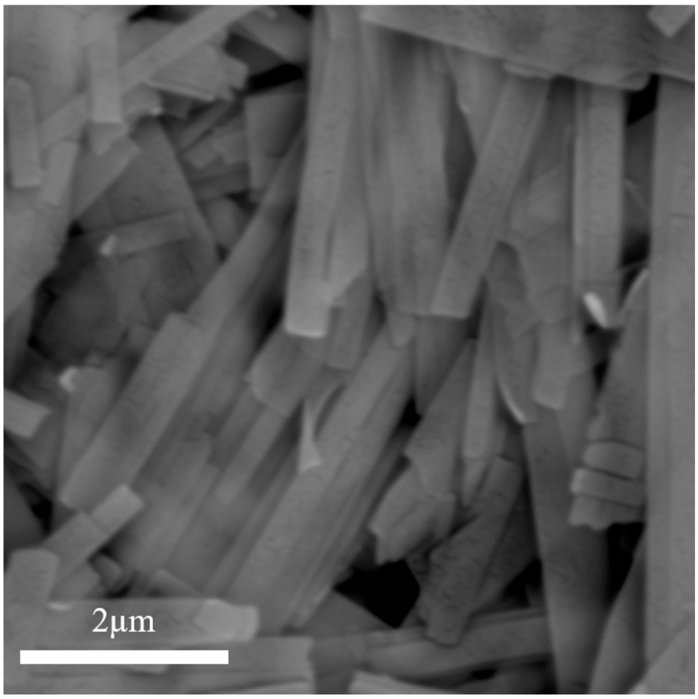

Figure S2. The SEM micrograph of the CVO cathode.

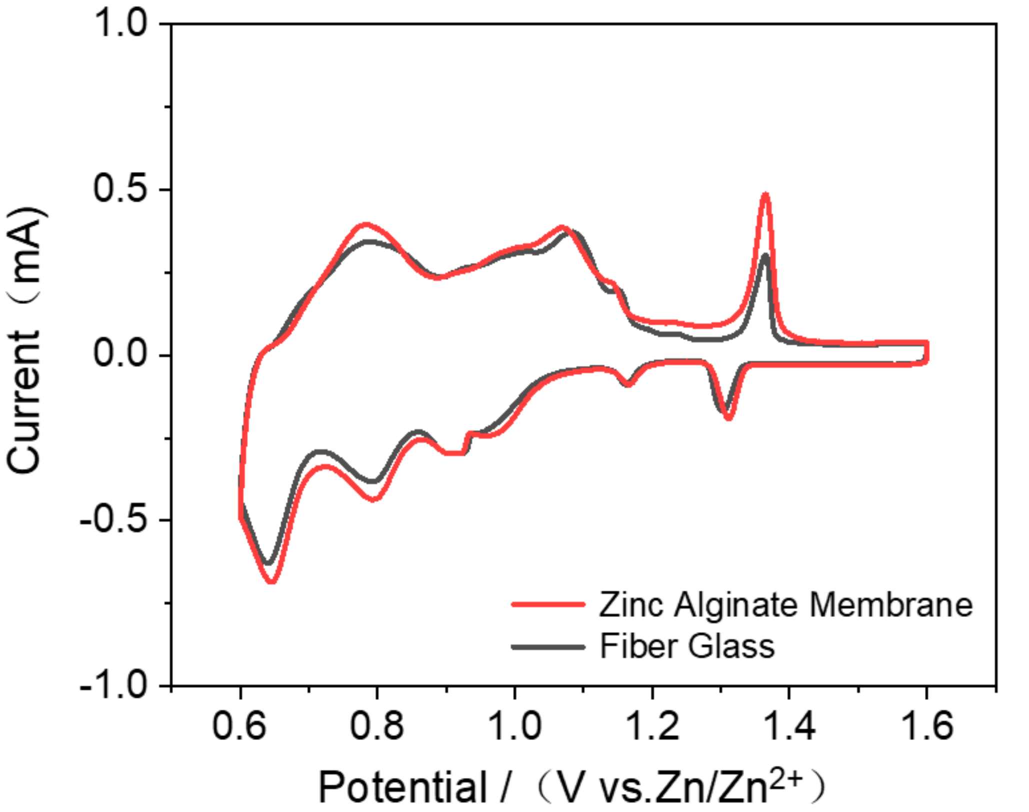

Figure S3. Cyclic voltammetry curves of Zn/ZA-based HGPE/CVO cell and Zn/wet glass fiber separator/CVO cell; scan rate: 0.2 mV s^{-1}.

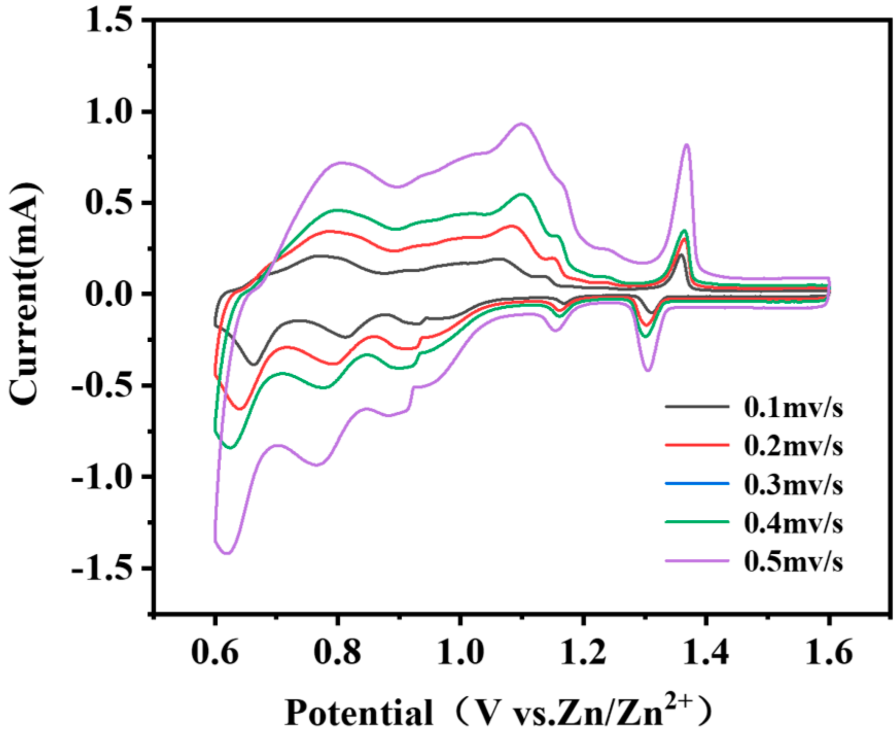

Figure S4. Cyclic voltammetry curves of Zn/ZA-based HGPE/CVO cell at different scan rates: 0.1-0.5 mV s^{-1}.

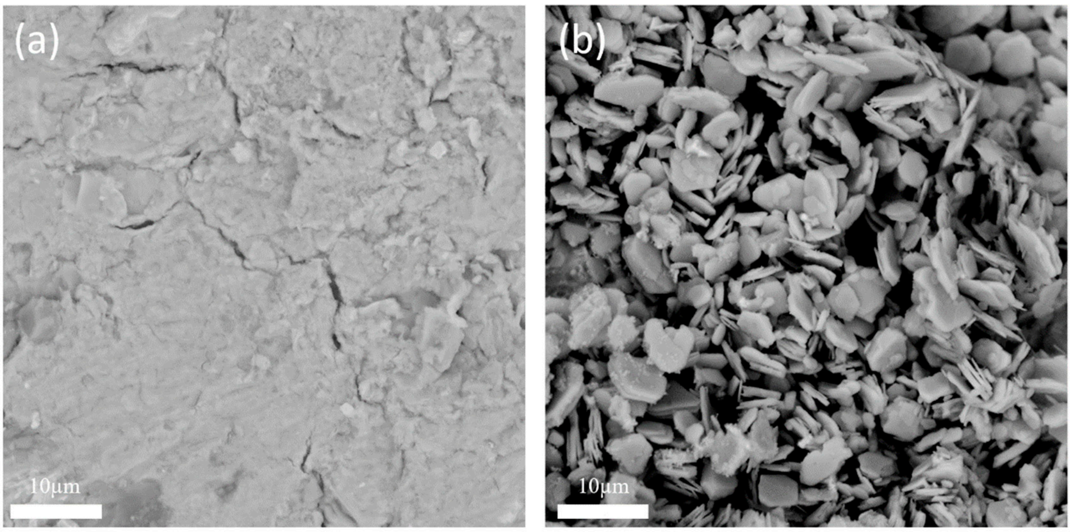

Figure S5. SEM micrographs of the Zn metal surfaces after 300 cycles at 3C in (a) Zn/ZA-based HGPE/CVO cell and (b) Zn/wet glass fiber separator/CVO cell.
